# Identification of Crowding Stress Tolerance Co-Expression Networks Involved in Sweet Corn Yield

**DOI:** 10.1371/journal.pone.0147418

**Published:** 2016-01-21

**Authors:** Eunsoo Choe, Jenny Drnevich, Martin M. Williams

**Affiliations:** 1 Global Change and Photosynthesis Research Unit, United States Department of Agriculture-Agricultural Research Service (USDA-ARS), Urbana, Illinois, United States of America; 2 Roy J. Carver Biotechnology Center, University of Illinois, Urbana, Illinois, United States of America; Institute of Genetics and Developmental Biology, Chinese Academy of Sciences, CHINA

## Abstract

Tolerance to crowding stress has played a crucial role in improving agronomic productivity in field corn; however, commercial sweet corn hybrids vary greatly in crowding stress tolerance. The objectives were to 1) explore transcriptional changes among sweet corn hybrids with differential yield under crowding stress, 2) identify relationships between phenotypic responses and gene expression patterns, and 3) identify groups of genes associated with yield and crowding stress tolerance. Under conditions of crowding stress, three high-yielding and three low-yielding sweet corn hybrids were grouped for transcriptional and phenotypic analyses. Transcriptional analyses identified from 372 to 859 common differentially expressed genes (DEGs) for each hybrid. Large gene expression pattern variation among hybrids and only 26 common DEGs across all hybrid comparisons were identified, suggesting each hybrid has a unique response to crowding stress. Over-represented biological functions of DEGs also differed among hybrids. Strong correlation was observed between: 1) modules with up-regulation in high-yielding hybrids and yield traits, and 2) modules with up-regulation in low-yielding hybrids and plant/ear traits. Modules linked with yield traits may be important crowding stress response mechanisms influencing crop yield. Functional analysis of the modules and common DEGs identified candidate crowding stress tolerant processes in photosynthesis, glycolysis, cell wall, carbohydrate/nitrogen metabolic process, chromatin, and transcription regulation. Moreover, these biological functions were greatly inter-connected, indicating the importance of improving the mechanisms as a network.

## Introduction

Modern corn (*Zea mays*) yields are largely the result of the interaction between improved genetics and agronomy [1–3]. Regarding genetics, tolerance to biotic and abiotic stresses has been significant [2]. Improvements in crowding stress tolerance have enabled the use of increasingly higher plant population density (‘plant density’) over the last 80 years [1]. Therefore yield of today’s hybrids are in part the result of high plant density made possible by crowding stress tolerance.

Tolerance to high plant density varies greatly among modern commercial sweet corn hybrids [4, 5]. Evaluation of 26 hybrids showed distinct yield differences when grown at high plant density [4]. Under conditions of crowding stress, high-yielding hybrids demonstrate greater crowding stress tolerance than low-yielding hybrids. Understanding and utilizing the genetic mechanism(s) that sustain yield under high plant density may provide a key target to future sweet corn improvement.

As plant density increases, individual plants experience crowding stress due to resource competition. Plant stress tolerance mechanisms can be defined as biological processes that reduce stress via resource capture and/or utilization [2]. Although plant biological responses to multiple abiotic stresses such as shade, water, and nutrients are critical components to crowding stress tolerance, plant responses to abiotic stress may not always influence yield. Studies found morphological changes of corn plants under crowding stress [6–8]. Increases in physiological processes such as nitrogen use efficiency [9], leaf photosynthesis rate [10], and post-flowering source-sink ratio [11] are possible factors in crowding stress tolerance. However, agronomically meaningful processes have a positive influence in crop yield potential [12]. Although shoot height and canopy leaf area index increase with crowding stress [6], these plant traits are not always associated with yield improvement. Moreover, manipulating biological processes to improve water-use efficiency are associated with reductions in biomass accumulation [13, 14]. In contrast, yield-related traits such as ear barrenness [15] or grain number per cob [16, 17] may be more direct indicators to crowding stress tolerance and yield. Identifying stress tolerance mechanisms linked with improving yield-related traits will be critical for agronomic utilization.

Transcriptional profiling has been a useful tool for understanding plant response to various abiotic stress factors in corn [18–20]. However, limited information is available on the relationships among transcriptional and phenotypic responses, particularly yield. Recent advances in transcriptional co-expression network analysis enables exploration of gene expression patterns with phenotypic and physiological traits in higher plants such as corn and *Arabidopsis* [21, 22]. Exploring transcriptional patterns among hybrids differing in yield under crowding stress, and connecting phenotypic responses with transcriptional responses, could identify mechanisms of crowding stress tolerance.

The goal of this work was to elucidate mechanisms conferring tolerance to crowding stress in sweet corn. Specifically, the objectives were to 1) explore transcriptional changes among sweet corn hybrids with differential yield under crowding stress, 2) identify relationships among phenotypic responses and gene expression patterns, and 3) identify groups of genes associated with yield and crowding stress tolerance.

## Materials and Methods

### Plant materials and field experiments

Yield data from twenty-six sweet corn hybrids evaluated in 2012 under crowding stress were used to identify crowding stress tolerant and sensitive germplasm [5]. Based on kernel mass per hectare, the three highest-yielding hybrids and three lowest-yielding hybrids were grouped into high- and low-yielding groups, respectively (Table 1). Average yield difference between groups was ~3 Mt per hectare. The high- and low-yielding groups represented crowding stress tolerant and sensitive groups, respectively.

**Table 1:** Description of six sweet corn hybrids used in the experiment.

Hybrid	Abbreviation	Seed source	Observed yield ^a^ Mt ha^-1^	Yield group
DMC21-84	H1	Del Monte Foods	9.39 a^b^	High
GG641	H2	General Mills	10.19 a	High
DMX22-90	H3	Del Monte Foods	9.71 a	High
GSS2259P	L1	Syngenta	6.50 b	Low
Magnum II	L2	Syngenta	6.69 b	Low
Rana	L3	Crookham Company	6.86 b	Low

^a^ Observed yield was measured from the field experiment conducted in 2012.

^b^ Hybrids with the same letters indicate the means are not significantly different at α = 0.05.

In 2013, a field experiment was conducted at the University of Illinois Vegetable Crop Research Farm near Urbana, IL. The six hybrids were grown at 71,700 plants per hectare, the optimal plant density of the most crowding stress-tolerant hybrid from the previous study and 13,700 plants per hectare above normal [4]. Production practices common to the region, such as tillage and pest control, were used. All plots were fertilized with 202 kg N per hectare prior to planting and sprinkler irrigated as needed to ensure crop establishment and maintain homogeneity of water and fertility throughout the field. The experimental design was a randomized complete block with 4 replications. Each plot consisted of four rows of each hybrid 9 m long with 76 cm spacing between rows. The experiment was planted at 65 plants per row and hand-thinned to 50 plants per row at 3-collar corn to achieve the target plant density. Stand counts were done after thinning and at harvest to confirm plant density.

Plant data were collected from the center two rows of each plot. Thermal time to mid-silk was determined as cumulative growing degree days (GDD) from crop emergence to mid-silk date. Plant height was measured on 6-collar corn (V6) and at silking (R1), taken from the soil surface to uppermost leaf apex. Leaf greenness was measured using a chlorophyll meter (SPAD 502 plus chlorophyll meter, Konica Minolta) for relative chlorophyll content on the mid-length of the oldest emerging leaf at V6 and R1. Leaf area index (LAI) was measured at R1 using a linear ceptometer (LP-80 AccuPAR, Decagon Devices, Pullman, WA). Total leaf nitrogen content was assessed from a sample of six primary ear leaves per plot. All measurements taken from multiple plants per plot were averaged by each plot for statistical analyses.

Approximately 21 days after mid-silk date of the plots, green ears (> 4.5 cm in diameter) were hand-harvested from the center two rows in 6.1 m length. Ear traits which are indirectly related with yield were measured on green ears. Ear length and filled ear length were measured from five random ears per plot and fill percentages were calculated by dividing mean filled ear length by mean ear length. Kernel moisture was calculated by the quantity fresh kernel mass minus dried kernel mass divided by fresh kernel mass times 100.

Yield traits were measured on harvested ears and kernels. Twelve random ears per plot were husked (A&K Development, Eugene, OR) and kernels were cut from the cob with an industry-grade hand-fed corn cutter (A&K Development, Eugene, OR). Green ear mass, husked ear mass, cob mass and kernel mass of 12 ear samples were measured. Based on these measurements, yield traits such as ear number per plant, ear number per hectare, ear mass per plant, ear mass per hectare, kernel mass per hectare, and kernel mass per plant was calculated.

### Statistical analysis of field data

Phenotypic data from field experiments were analyzed using PROC MIXED in SAS version 9.2 (SAS Institute Inc. Cary, NC, USA). Yield group and hybrids nested within group were considered fixed factors. Replications were considered random factors. Data complied with ANOVA assumptions of homogeneity of variance based on the modified Levene’s test [23] and normality based on diagnostic test of residuals.

### Plant materials and microarray experiment

Plant tissue samples were collected by bulking 4 ear leaves per plot at the R1 growth stage. The reproductive stage of the plant was selected because corn is most susceptible to stress at flowering, when silk growth, pollination, and kernel set occur [24]. Moreover, leaf photosynthesis after anthesis influences biomass accumulation and allocation in corn under crowding stress [25]. Two to three biological replications per hybrid were frozen in liquid nitrogen immediately after removal from the plant and stored at -80 C until RNA extraction.

Total RNA was extracted from 16 samples (H1, H2, L1 and L2 with 3 replications; H3 and L3 with 2 replications) using the RNeasy mini kit (Qiagen, Hilden, North Rhine-Westphalia, Germany). Quantity and quality of total RNA was checked using the Agilent 2100 Bioanalyzer (Agilent Technologies, Inc. USA). A microarray experiment was performed at the Roy J. Carver Biotechnology Center at University of Illinois. The microarray design was based on field corn inbred B73 coding sequences from MaizeGDB (http://www.MaizeGDB.org). A set of gene representations was created by retaining the longest transcript from each gene. A custom microarray was designed using Agilent eArray (Agilent Amadid # 060449). Out of 39,653 coding sequences, 39,091 unique probes were designed. The array contained 39,091 unique probes, of which 34,379 were single-spotted and 4,712 were double-spotted, plus 1,264 positive controls and 153 negative controls. Seventy-five ng of total RNA was labeled using the Agilent 2-color Low Input Quickamp Whole Transcriptome Labeling kit (Agilent Technologies, Santa Clara, CA) according to manufacturer protocol. Labeled samples were hybridized on the custom microarray (4x44K format) and scanned on an Axon 4000B microarray scanner (Molecular Devices, Sunnyvale, CA) at 5 um resolution. GenePix 6.1 image analysis software (Molecular Devices, Sunnyvale, CA) was used for spotfinding.

### Statistical analyses of microarray data

Pre-processing and statistical analyses of microarray data were performed in R (version 3.2.1) using the Limma package [26] (version 3.24.15). Median foreground values from the 8 arrays were read into R and any spots that had been manually flagged (-100 values) were given a weight of zero [27]. The individual Cy5 and Cy3 values were all normalized together using the quantile method and then log2-transformed [27]. Correlations between replicate spots for the double-spotted probes were high; therefore, expression values were averaged to obtain a single value for the double-spotted probes for each sample. The positive and negative control probes were used to assess the minimum expression level considered ‘detectable above background noise’ (6.25 on the log2 scale) and then discarded.

A mixed effects statistical model [28] was fit on the 39,091 unique probes to estimate the mean expression level for each of the 6 hybrid groups while accounting for the random effect of array [29]. After model fitting, 8,901 probes were discarded because they did not have expression values > 6.25 in at least 2 samples. For ease of discussion, the remaining 30,190 probes will be called genes. Principal component analysis (PCA) was performed on 30,190 genes to examine the group and hybrid effects on overall gene expression.

From the group means model, a contrast of all high-yielding hybrids vs. all low-yielding hybrids was made. Since hybrid effect was significant, pairwise hybrid comparisons were made between each hybrid from high- versus low-yielding groups to identify individual hybrid differences. Fold change (FC) from pairwise comparisons were calculated to show up- or down-regulation of genes between high- and low-yielding hybrids. Raw p-values were adjusted separately for each comparison using the False Discovery Rate (FDR) method [30]. Differentially expressed genes (DEGs) were identified from pairwise comparisons when FDR p<0.05 and |FC|>1.5.

Additionally, a one-way ANOVA across all 6 hybrids was computed to identify genes that changed among the 6 hybrids. From the result, 7,670 genes were identified that had FDR p-value < 0.05 and |FC|>1.5 between any two hybrid comparisons. Weighted Gene Correlation Network Analysis (WGCNA) [31, 32] was performed using these 7,670 genes to cluster genes using a distance metric that separates them into different ‘modules’ which share a consistent expression pattern associated with phenotypic responses. The genes were clustered using WGCNA (version 1.47) package using the default values of the blockwiseModules() function except for: soft thresholding power β = 30, networkType = ‘signed’, minModuleSize = 20, and mergeCutHeight = 0.25. This resulted in 40 modules ranging from 30 to 679 genes, plus the ‘module 0’ consisting of 6 genes that did not fit any of the other patterns. The overall expression pattern of each module can be represented by the eigengene values, which are the first principal component score for each sample from the expression values of the genes in the module. The eigengene values for each module were then subjected to an overall high-yielding versus low-yielding group comparison in order to determine modules related to group differences.

Functional analysis and visualization of groups of DEGs and modules were conducted using AgriGO [33], REVIGO [34], Cytoscape (version 3.2.1) [35], and Mapman software [36]. Over-representation analysis was conducted using Fisher’s exact test based on corn gene ontology (GO) and Mapman annotation. All p-values were adjusted using Benjamini Hochberg FDR method with significance level at 0.05 [30].

### Validation of gene expression using RT-qPCR

Quantitative reverse transcription-polymerase chain reaction (RT-qPCR) was performed to validate microarray results. Four transcripts were selected based on their importance to crowding stress response from this microarray experiment and previous studies (S1 File) [19, 37]. Ubiquitin conjugating enzyme was selected as the endogenous control. Invitrogen Superscript First-Strand Synthesis System (Invitrogen) was used to synthesize cDNA from the same total RNA samples. Primer Express Software Version 3.0 (Applied Biosystems, Foster, CA) was used to design primers. Power SYBR Green Master Mix (Applied Biosystems, Foster, CA) and ABI 7900 real time PCR machine were used for performing RT-qPCR. SDS2.4 software (Applied Biosystems, Foster, CA) was used to analyze the RT-qPCR result and identify threshold values. Three technical replications were used for each sample and averaged for the analysis when PCR efficiency was between 90 and 100% and R^2^ close to 0.99 [38]. The cycle threshold values were normalized to the expression of control genes and the ∆∆Ct method was used for comparing the gene expression values involved in crowding stress [38].

## Results and Discussion

### Phenotypic response under crowding stress

For most yield traits, higher values were observed in the high-yielding group than the low-yielding group (Table 2). For instance, ear mass per hectare and kernel mass per hectare were 26% and 90% higher in the high-yielding group, respectively. Hybrids within a yield group were similar for all yield traits. All hybrids produced less than 1 ear per plant, indicating plants experienced crowding stress.

**Table 2:** Phenotypic traits of sweet corn hybrids and yield groups measured in a field experiment near Urbana, IL in 2013.

	Plant traits					Ear traits				Yield traits					
Effect	Plant height at V6	Plant height at R1	SPAD^b^ at V6	SPAD at R1	GDD^c^ to silk	Ear length	Fill length	Fill percentage	Kernel moisture	Number of ear plant^-1^	Ear mass plant^-1^	Kernel mass plant^-1^	Number of ears ha^-1^	Ear mass ha^-1^	Kernel mass ha^-1^
	cm	cm	-	-	-	cm	cm	%	%	No. plant^-1^	Kg plant^-1^	g plant^-1^	No. ha^-1^	Mt ha^-1^	Mt ha^-1^
**Hybrid**															
H1	44.8 a^a^	190.4 c	47.6 bc	55.0 ab	739 c	16.6 c	15.7 b	94.7 ab	76.7 c	0.88 a	0.30 a	124.7 a	66,198 a	23.0 a	9.42 a
H2	46.3 a	201.8 b	46.5 bc	54.0 ab	744 c	17.7 bc	15.1 b	85.7 cd	77.4 bc	0.92 a	0.32 a	139.9 a	64,315 ab	22.2 a	9.80 a
H3	45.9 a	188.8 c	45.4 c	55.5 ab	727 c	19.4 a	16.3 ab	84.0 d	77.1 c	0.85 ab	0.30 a	126.2 a	65,391 a	23.1 a	9.73 a
L1	44.5 a	223.0 a	49.4 ab	55.3 ab	822 a	17.8 bc	16.1 ab	90.5 bc	83.9 a	0.82 ab	0.25 b	63.0 b	57,049 ab	17.8 b	4.41 b
L2	41.6 a	197.6 bc	48.5 bc	49.2 b	789 b	18.3 ab	17.1 a	93.2 ab	77.0 c	0.74 b	0.24 b	73.8 b	53,820 b	17.2 b	5.35 b
L3	41.8 a	161.0 d	53.3 a	57.2 a	693 d	16.7 c	16.3 ab	97.5 a	79.0 b	0.83 ab	0.26 b	72.7 b	62,162 ab	19.4 b	5.44 b
**Yield group**															
High	45.6 a	193.7 a	46.5 b	54.8 a	736 b	17.9 a	15.7 b	88.1 b	77.1 b	0.88 a	0.31 a	130.2 a	65,301 a	22.8 a	9.65 a
Low	42.6 b	193.9 a	50.4 a	53.9 a	768 a	17.6 a	16.5 a	93.7 a	80.0 a	0.80 a	0.25 b	69.8 b	57,677 b	18.1 b	5.07 b

^a^ Mean comparisons were performed among hybrids and between groups. For each effect, the same letters within a column indicate the means are not significantly different at α = 0.05.

^b^ Measurement of leaf greenness.

^c^ Cumulative growing degree days.

Effects of yield group and hybrid were significant for most plant and ear traits, but not all (Table 2). Hybrid L1 was the tallest plant at R1 (223.0 cm) and latest maturity (822 GDD to silk) hybrid. In contrast, hybrid L3 was the shortest plant at R1 (161.0 cm) and earliest maturity (693 GDD to silk) hybrid. Of ear traits, H3 had the largest ear length but the smallest fill percentage. Leaf nitrogen and LAI were similar among hybrids, averaging 2.89% and 5.53, respectively (S3 File).

Kernel mass per hectare was positively correlated with all yield traits (ρ ≥ 0.46) and plant height at V6 (ρ = 0.29). Kernel moisture and fill percentage were negatively correlated (ρ≤ -0.52) with kernel mass per hectare (Table 3).

**Table 3:** Correlation coefficients between phenotypic responses and kernel mass per hectare.

Response type	Phenotypic response	Correlation coefficients
Plant traits	Plant height at V6	0.29 *^a^
	Plant height at R1	0.04
	SPAD at V6	-0.26
	SPAD at R1	0.12
	GDD to silk	-0.22
	LAI	-0.02
	Leaf nitrogen	-0.25
Ear traits	Kernel moisture	-0.55 ***
	Ear length	0.23
	Fill length	-0.04
	Fill percentage	-0.52 ***
Yield traits	Number of ear plant^-1^	0.46 **
	Ear mass plant^-1^	0.79 ***
	Kernel mass plant^-1^	0.98 ***
	Number of ears ha^-1^	0.57 ***
	Ear mass ha^-1^	0.86 ***

^a^ Correlation significant at

*, α = 0.05

**, α = 0.01

***, α = 0.001.

Most phenotypic responses varied among sweet corn hybrids, indicating hybrids were genetically different in their response to crowding stress. In contrast, yield trait differences among hybrids within yield group were consistent (Table 2) indicating common tolerance mechanism(s) may be present in high-yielding hybrids.

Agronomically useful crowding stress tolerance mechanisms should be associated with greater yield potential. Plant responses such as plant height and SPAD were significant at V6, but not significant at R1 between yield groups. Difference in plant height and SPAD at V6 may be important indicators to plant competition during vegetative growth stage. However, no differences at R1 suggest plant height and SPAD lack consistent connections with yield at the flowering stage. Difference in GDD to silk between yield groups was due largely to late maturity in L1 and L2. Hybrid L3 had the earliest maturity. Therefore GDD to silk may not be suitable for discriminating yield groups. Identifying internal genetic mechanisms that explain yield group differences would be critical for better understanding of crowding stress tolerance.

### Transcriptional difference between crowding stress tolerant and sensitive groups

The microarray experiment was performed to compare gene expression patterns among hybrids. Results have been submitted in NCBI’s Gene Expression Omnibus database [39] and are accessible through GEO Series accession number GSE73435 (http://www.ncbi.nlm.nih.gov/geo/query/acc.cgi?acc=GSE73435). The microarray result was validated using RT-qPCR (S1 and S2 Files).

Initial investigation of gene expression patterns showed a strong hybrid effect, yet little similarity within the same yield group. The heatmap showed varied expression patterns across hybrids (S1 Fig). Principal component analysis (PCA) and hierarchical clustering indicated L1 was most different from all other hybrids (S2 Fig). However, there were no clear visual patterns or clusters of transcripts to differentiate yield groups.

Out of 39,091 transcripts assayed, 199 up-regulated and 207 down-regulated DEGs in the high-yielding group were identified from the contrast of all high-yielding hybrids vs. all low-yielding hybrids (Data not shown). Nonetheless, understanding individual hybrid gene expression patterns to crowding stress may be more important than averaging expression across hybrids.

**Fig 1:**
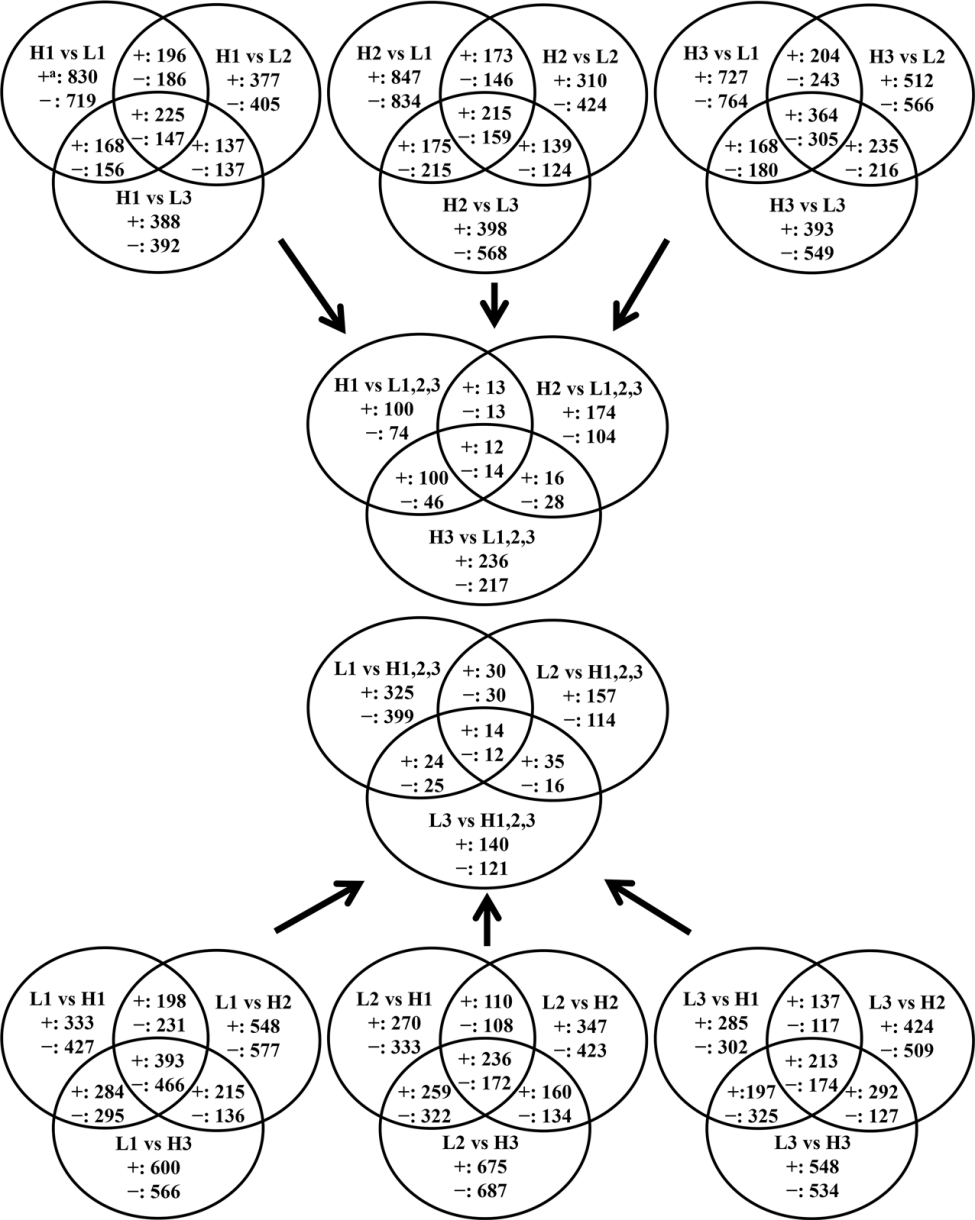
Venn diagrams to identify common DEGs from pairwise comparisons. ^a^ Up- and down- regulated DEGs in the first hybrid from listed pairwise comparison identified at FDR p-value<0.05 and |FC|>1.5 were labeled + and -, respectively.

Pairwise comparisons between hybrids were conducted to identify common DEGs. For each hybrid, groups of DEGs were first identified from pairwise comparisons. Common DEGs among three pairwise comparisons of one high-yielding hybrid to all low-yielding hybrids (e.g. H1 vs. L1, L2 and L3) were identified and vice versa. These common DEGs represented unique genes influenced by crowding stress of each high-yielding hybrid. There were 372 (225 up- and 147 down-regulated), 374 (215 up- and 159 down-regulated), and 669 (364 up-and 305 down-regulated) common DEGs in H1, H2 and H3 compared to all low-yielding hybrids, respectively (Fig 1). Likewise 859 (393 up- and 466 down-regulated), 408 (236 up- and 172 down-regulated) and 387(213 up- and 174 down-regulated) DEGs were identified in L1, L2 and L3 compared to all high-yielding hybrids. There were 14 (up-regulated in high-yielding hybrids) and 12 (down-regulated in high-yielding hybrids) common DEGs across all pairwise comparisons, accounting for only 3 to 7% of common DEGs (Fig 1). The small number of common DEGs was identified across all pairwise comparisons implies each hybrid has a unique mechanism(s) in crowding stress response.

Functional analysis of common DEGs revealed distinct functional differences among hybrids (Table 4). A previous transcriptional study on density stress on corn seedlings found a small number of genes commonly expressed among different genotypes, indicating genotype is an important factor to crowding stress response [19]. Plants under crowding stress experience growth alterations due to differences in individual plant resource requirement and/or resource utilizing ability [2]. Different genotypes may experience varying levels of crowding tolerance even in similar environmental conditions (i.e. same plant density). Therefore, high-yielding hybrids have genetic mechanisms in tolerating crowding stress and resulting in better kernel and ear mass than low-yielding hybrids. For example, biological functions identified from high-yielding hybrids such as genes involved in steroid biosynthetic process (H1) [40], fatty acid/carbohydrate metabolic process (H2) [40, 41] or GTP/UTP/CTP biosynthetic process (H3) [42] may have associations with yield gain (Table 4). In contrast, low-yielding hybrids are more sensitive to crowding stress than high-yielding hybrids that resulted in reduction in kernel and ear mass. Biological functions such as genes involved in sexual reproduction/cell wall organization (L1) [43], electron transport chain/cytochrome complex assembly (L2) [44], or apoptotic process/oligopeptide transport (L3) [45] may be associated with crowding stress sensitivity (Table 4). Collectively, this genomic information indicates that each hybrid has a unique crowding stress response mechanism. Significant biological functions identified in all hybrids may be involved in crowding stress response, but the function identified in high-yielding hybrids will have a positive association with yield. These mechanisms of crowding stress tolerance in high-yielding hybrids may be exploited for further agronomic improvement.

**Table 4:** Over-represented biological processes of common DEGs identified for each hybrid.

Hybrid	Gene ontology (GO) terms (Biological function)	FDR p-value
H1	Steroid biosynthetic process	0.063
H2	Metabolic process	<0.001
	Fatty acid biosynthetic process	0.022
	Carbohydrate metabolic process	0.048
H3	GTP, UTP, CTP biosynthetic process	0.002
	Glutamine metabolic process	0.045
L1	Sexual reproduction	<0.001
	Plant type cell wall organization	<0.001
	Carbohydrate metabolic process	0.012
	Glutamate biosynthetic process	0.025
L2	Electron transport chain	0.003
	Cytochrome complex assembly	0.025
	tRNA splicing via endonucleolytic cleavage and ligation	0.046
L3	Apoptotic process	0.001
	Oligopeptide transport	0.040

Of 26 DEGs significant among all pairwise hybrid comparisons, 15 DEGs related to known biological functions. Up-regulated DEGs in high-yielding hybrids were involved in amino acid degradation/polyamine metabolism, cell wall degradation, glycolysis cytosolic branch, hormone metabolism (Auxin), RNA transcription regulation, signaling receptor kinases and tetrapyrrole synthesis. Whereas down-regulated DEGs in high-yielding hybrids were involved in flavonoids secondary metabolism, miscellaneous enzyme families, protein folding, protein post-translational modification and development (Table 5). These genes may be important candidate genes linked to crowding stress tolerance.

**Table 5:** The description of common DEGs identified from all pairwise comparisons.

ID	FC^a^	MapMan description	Associated enzymes or genes
grmzm2g374302_t02	UP	Amino acid degradation/polyamine metabolism synthesis	Arginine decarboxylase1 (ADC1) (EC 4.1.1.19)
grmzm2g151257_t01	UP	Cell wall degradation	Cellulases /Endoglucanase 1 (EC 3.2.1.4)
ac205100.3_fgt001	UP	Glycolysis cytosolic branch	Pyrophosphate-fructose-6-P phosphotransferase (EC 2.7.1.90)
grmzm2g431291_t01	UP	Hormone metabolism (Auxin)	-
grmzm5g854473_t01	UP	RNA transcription regulation	BolA-like family protein
grmzm5g889999_t01	UP	Signaling receptor kinases	Kinase interacting kinase 1 (kik1), B120
grmzm2g043277_t02	UP	Tetrapyrrole synthesis	Heme oxygenase, TED4
ac234526.1_fgt005	DOWN	Flavonoids secondary metabolism	Dihydroflavonol-4-reductase (DFR) (EC 1.1.1.219)
grmzm2g008935_t01	DOWN	Miscellaneous enzyme families	Flavonol 3-O-glucosyl transferase
grmzm2g151332_t01	DOWN	Miscellaneous enzyme families	Flavonol 3-O-glucosyl transferase
grmzm2g161625_t02	DOWN	Miscellaneous enzyme families	Flavonol 3-O-glucosyl transferase, UGT73D1
grmzm2g170047_t01	DOWN	Miscellaneous enzyme families	Indoleacetaldoxime dehydratase, CYP71A25
grmzm2g068963_t01	DOWN	Protein folding	FK506 binding protein
ac197029.3_fgt003	DOWN	Protein post-translational modification	*Arabidopsis* NPK1-related protein kinase 3 (ANP3)
grmzm2g114751_t01	DOWN	Development	-

^a^ Fold changes (FC) were labeled UP for higher expression in all high yielding hybrids versus all low yielding hybrids, and DOWN for lower expression in all high yielding hybrids versus all low yielding hybrids.

### Connecting phenotypic traits and transcript expression

In addition to individual gene differences, constructing a co-expression network of transcriptome data is increasingly used to understand complex gene interactions and to correlate gene expression patterns to phenotypic traits of crops [21, 22, 46–48]. We performed WGCNA in order to identify relationships among phenotypic responses and gene expression patterns of groups of genes associated with yield and crowding stress tolerance. The eigengene values of 40 modules were tested for group difference, which identified 9 significant modules. These 9 modules consisted of 51 to 264 genes. On average, modules 10, 13, 32, 36 and 38 showed up-regulation and modules 9, 14, 17 and 22 showed down-regulation in the high-yielding group (S3 Fig). Only modules 22 and 36 showed the same expression patterns in all hybrids within a group. Remaining modules showed the same expression in two out of three hybrids in a group.

**Fig 2:**
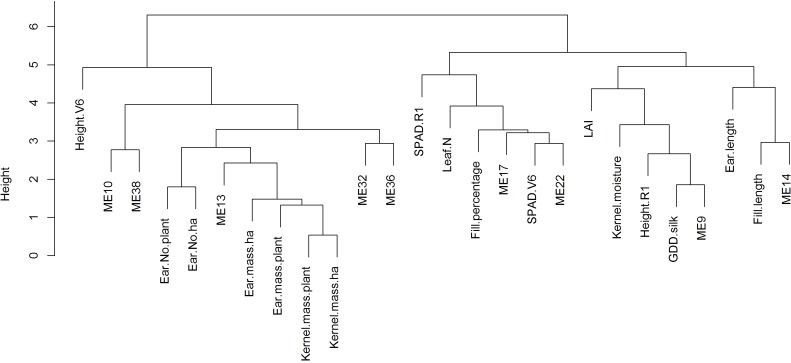
Hierarchical clustering of phenotypic traits and module eigengene values (ME). Phenotypic traits were labeled as ‘Height.V6’, height at V6; ‘Ear.No.plant’, number of ears per plant; ‘Ear.No.ha’, number of ears per hectare; ‘Kernel.mass.plant’, kernel mass per plant; ‘Kernel.mass.ha’, kernel mass per hectare; ‘SPAD.R1’, SPAD at R1; ‘Leaf.N’, leaf nitrogen; ‘Fill.percentage’, fill percentage; ‘SPAD.V6’, SPAD at V6; ‘LAI’, leaf area index; ‘Kernel.moisture’, kernel moisture; ‘Height.R1’, height at R1; ‘GDD.silk’, GDD to silk; ‘Ear.length’, ear length; ‘Fill.length’, fill length.

Correlation analysis among these 9 modules and phenotypic traits showed positive associations of up-regulated modules with yield traits but negative associations of down-regulated modules with yield traits (Table 6). In contrast, most plant and ear traits were not correlated with up-regulated modules. Notably, GDD to silk, fill percentage, and SPAD at V6 were negatively correlated with module 13, 32, and 36, respectively. These three plant and ear traits were positively correlated with down-regulated modules 9, 14 and 22, respectively (Table 6). Hierarchical clustering of modules and phenotypic traits showed two large branches. One cluster consisted of the up-regulated modules and yield traits, and the other cluster contained the down-regulated modules, plant and ear traits (Fig 2). Positive correlation and clustering of up-regulated modules with yield traits also support that these modules have positive genetic effects on yield.

**Table 6:** Correlation coefficients between phenotypic traits and modules with significant expression differences between high- and low-yielding groups. Correlation coefficients with bold text significant at FDR p-value<0.05.

		UP					DOWN			
Response type	Phenotypic response	Module 10	Module 13	Module 32	Module 36	Module 38	Module 9	Module 14	Module 17	Module 22
Plant traits	Plant height at V6	-0.06	0.11	0.14	0.21	0.28	-0.16	-0.44	-0.01	-0.27
	Plant height at R1	-0.21	-0.58	-0.19	0.17	0.30	0.61	-0.14	0.29	-0.11
	SPAD at V6	-0.52	-0.15	-0.55	**-0.78**	-0.47	0.04	0.05	0.62	**0.71**
	SPAD at R1	0.05	0.51	0.05	-0.35	0.13	-0.48	-0.55	0.13	0.09
	GDD to silk	-0.24	**-0.83**	-0.42	-0.08	0.02	**0.88**	0.26	0.43	0.20
	LAI	-0.15	-0.34	-0.28	-0.43	-0.15	0.39	0.13	0.32	0.38
	Leaf nitrogen	0.10	-0.03	-0.50	-0.40	-0.05	0.15	0.02	0.49	0.34
Ear traits	Ear length	0.13	-0.23	0.43	0.32	0.04	0.24	0.24	-0.48	-0.26
	Fill length	0.08	-0.44	-0.35	-0.35	-0.40	0.54	**0.71**	0.16	0.44
	Fill percentage	-0.04	-0.08	**-0.71**	-0.63	-0.35	0.15	0.29	0.62	0.62
	Kernel moisture	-0.33	-0.51	-0.44	-0.48	0.07	0.55	-0.23	0.63	0.36
Yield traits	Number of ear plant^-1^	0.18	0.64	0.47	0.43	0.48	-0.65	**-0.68**	-0.32	-0.56
	Ear mass plant^-1^	0.44	**0.78**	**0.75**	**0.79**	**0.68**	**-0.74**	**-0.70**	-0.65	**-0.89**
	Kernel mass plant^-1^	0.44	**0.76**	**0.77**	**0.85**	0.56	**-0.76**	-0.52	**-0.73**	**-0.88**
	Number of ears ha^-1^	0.33	**0.74**	0.38	0.26	0.40	**-0.69**	-0.57	-0.28	-0.43
	Ear mass ha^-1^	0.56	**0.87**	**0.68**	0.66	0.62	**-0.78**	-0.63	-0.60	**-0.79**
	Kernel mass ha^-1^	0.50	**0.80**	**0.74**	**0.81**	0.56	**-0.77**	-0.51	**-0.72**	**-0.86**

Enrichment analysis identified over-represented GO terms for each module. The most significant GO biological processes or molecular functions identified on up-regulated modules were cellular component organization (module 10), cellular nitrogen compound metabolic process/carbohydrate metabolic process (module 13), transcription regulation (module 32), and RNA splicing (module 36) (Table 7). The most significant GO terms identified on down-regulated modules were transporter activity/binding (module 9), translation (module 17), and metabolic process/Chitin catabolic process (module 22). Biological functions of genes and modules for each hybrid or group in this research provide useful genetic information on yield response to crowding stress.

**Table 7:** Over-represented biological processes of modules significant between high- and low-yielding groups. Biological functions for modules were significant at FDR p-value<0.05.

Module	FC	Number of genes	Gene ontology (GO) terms	Associated enzymes or genes
Module 10	UP	248	Cellular component organization	Histone H2B, H4
Module 13	UP	221	Cellular nitrogen compound metabolic process, carbohydrate metabolic process	Ferredoxin, light harvesting chlorophyll protein complex, heme oxygenase (grmzm2g043277_t02^a^), cellulases (grmzm2g151257_t01)
Module 32	UP	84	Transcription regulation	EREB67, auxin responsive protein IAA17, bZIP118, bZIP71, MADS19
Module 36	UP	66	RNA splicing	ADC1(grmzm2g374302_t02), Pyrophosphate-fructose-6-P phosphotransferase (ac205100.3_fgt001), auxin metabolism related (grmzm2g431291_t01), kik1(grmzm5g889999_t01)
Module 38	UP	51	-	-
Module 9	DOWN	264	Transporter activity/binding	-
Module 14	DOWN	220	-	-
Module 17	DOWN	198	Translation	-
Module 22	DOWN	156	Metabolic process/Chitin catabolic process	DFR (ac234526.1_fgt005), Flavonol 3-O-glucosyl transferase (grmzm2g008935_t01, grmzm2g151332_t01, grmzm2g161625_t02), FK506 binding protein (grmzm2g068963_t01), ANP3 (ac197029.3_fgt003), grmzm2g114751_t01

^a^ Transcript ID of common DEGs identified from all pairwise comparisons.

### Photosynthesis related metabolism

Reduction in photosynthate supply after anthesis was a critical cause of ear or kernel abortion under crowding stress [49, 50]. From pairwise comparisons, genes involved in electron transport chain were significant in L2. Most of down-regulated genes in electron transport chain in L2 are involved in light reaction with the highest FC in multiple ferredoxin electron transport related genes such as *ferredoxin 1* and *2*. Photosynthetic electron transport has an important function in metabolizing energy sources NADPH and ATP. Ferredoxin is a light-sensitive electron carrier [51] that has an important role in the multiple metabolic pathway regulations and transduction of redox signals into the regulatory network [52] and significantly associated with linear electron flow and photosynthesis capacity [53, 54]. Down-regulation or knockout of ferredoxin in tobacco, potato and *Arabidopsis* has resulted in growth inhibition and photosynthesis inactivation [53–56]. Studies also identified ferredoxin plays an important role in tolerance to many stress factors such as water deprivation, oxidative stress, and heat or cold stress [44]. Relative down-regulation of photosynthesis electron transport in L2 to all high-yielding hybrids may be part of a mechanism responsible for low productivity of L2 under crowding stress.

Genes involved in tetrapyrrole biosynthesis, especially in chlorophyll and heme, were up-regulated in all high-yielding hybrids. Heme oxygenase (grmzm2g043277_t02), required for phytochrome chromophore biosynthesis as a plant photoreceptor, was up-regulated in all high-yielding hybrids. *Arabidopsis* HY1 gene encoding heme oxygenase has been characterized and requires electron transfer from ferredoxin in vivo [57, 58]. These results suggest the critical and close interaction of photosynthesis-related biological processes, specifically electron transport and tetrapyrrole synthesis, may contribute to yield difference under crowding stress. The down-regulation of both ferredoxin and terapyrrole activities in L2 may provide the connection for significantly lower leaf chlorophyll content (SPAD) at R1 in L2 than other hybrids.

Important crowding stress tolerant mechanisms may be plant biological processes influencing yield. Stress tolerance mechanisms have to be evaluated for yield effect since mechanisms can be negatively associated with biomass accumulation [12–14]. Comparing DEGs among hybrids under crowding stress would not capture all crowding stress response genes since some stress response genes might be expressed similarly across all hybrids. For example, plant phytochrome photoreceptor genes such as phyA and phyB are important candidate genes involved in shade avoidance response due to reduction in the ratio of red:far-red light [37, 59]. Due to plant competition for light under crowding stress, these genes also may be involved in crowding stress response. However, five phyA and phyB related genes screened in the present work were expressed similarly across all hybrids. Therefore, although these genes may be involved in stress response mechanisms, they may not be important for crowding stress tolerance mechanisms influencing yield.

### Cell wall metabolism

Cell wall metabolism is a complex network responsible for various abiotic stress responses [60]. Transcript grmzm2g151257_t01, known for cellulases/endoglucanase (cell wall degradation), was up-regulated in all high-yielding hybrids. Cellulases are cell-wall-loosening protein genes involved in cell expansion and root elongation in *Arabidopsis* and believed to be important for drought tolerance [61] and corn yield [62]. Cell wall degradation activity is often closely regulated by plant hormones. Plant hormones have an important role in plant growth such as regulating the expression of root cell-wall-loosening protein genes [63, 64]. Plant hormone auxin is believed to regulate cellulase activity [65]. Up-regulation of auxin and jasmonate synthesis-related genes along with up-regulation of cellulase-related genes in all high-yielding hybrids suggests their role in crowding stress tolerance. Understanding the relationships of genes with different functions may be valuable for further investigation, because complex traits like corn yield are associated with a large chain of metabolic responses [66].

Other cell wall degradation and modification-related genes showed mixed expressions among hybrids. For example, many genes in cell wall degradation and modification were significantly down-regulated in L1. Out of 52 genes involved in cell wall degradation in L1, 20 common DEGs were identical or similar to corn, rice or *Arabidopsis* expansin, a protein involved in cell wall-loosening and cell growth [67]. Expansin showed mixed expression depending on plant species, varying in response to abiotic factors such as temperature, light, and water [60]. Expression of expansin was associated with plant growth and flowering date in rice [68]. Hybrid L1 showed a unique phenotypic response with the tallest plant at R1 and the latest maturity. Significant expression of expansin in L1 may have an influence in the phenotypic response.

### Primary and secondary metabolic pathway

Primary and secondary metabolic pathways such as glycolysis metabolism and polyamine synthesis were important in crowding stress tolerance. Glycolysis had a positive association with corn yield [62]. Most enzymes involved in cytosolic branch of glycolysis and mitochondrial electron transport were up-regulated in high-yielding hybrids, and the highest up-regulation was observed on pyrophosphate-fructose-6-P-phsphotransferase (ac205100.3_fgt001). This enzyme is involved in conversion of fructose-6-phosphate to fructose-1,6-bisphosphate. Increased activity of the enzyme has been observed in anaerobiosis condition in rice [69] and was positively associated with corn yield [62].

Arginine decarboxylase (grmzm2g374302_t02) was up-regulated in high-yielding hybrids. Arginine decarboxylase in *Arabidopsis* is a key enzyme involved in the first step of polyamine synthesis, converting arginine to agmatine. The enzyme influences early seed development during embryo growth. Arginine decarboxylase in *Arabidopsis* showed close relationship with the production of putrescine, one of the most abundant polyamines in plants and animals [70]. Polyamines have critical functions in senescence, cell proliferation, differentiation biotic and abiotic stress response [71–73]. Overexpression of arginine decarboxylase in tobacco and tomato was involved in drought tolerance by reactive oxygen species detoxification [74]. Arginine decarboxylase may be an important key to crowding stress tolerance in sweet corn.

Flavonoids are color pigments in plants, protecting leaf cells from photooxidative damages and improving the efficiency of nutrient retrieval during senescence [75]. Flavonol 3-O-glucosyltransferases is an enzyme involved in anthocyanin biosynthesis by providing sugars through UDP-glucose. Gene (Gdi-15), homologous to flavonol 3-O-glucosyltransferases, in groundnut responded to heat and moisture stress during early growth stage [76]. Three forms of flavonol 3-O-glucosyltransferases were down-regulated in high-yielding hybrids and all belonged to module 22, which was negatively correlated to yield traits and positively correlated to SPAD at V6. This relationship indicates the enzyme may have an important stress tolerance function during early vegetative stage, but may not be associated with yield.

**Fig 3:**
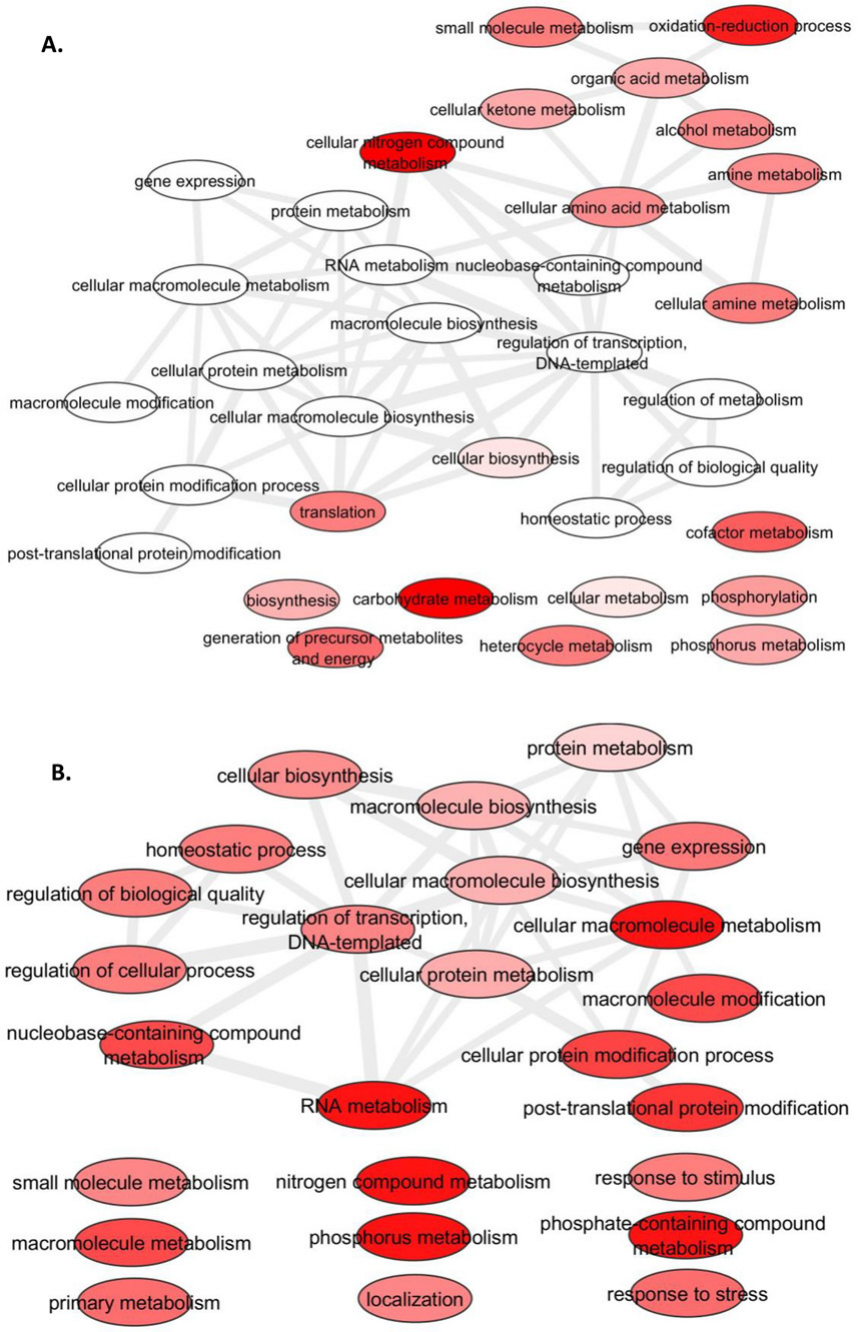
Visualization of GO terms in biological functions of module 13 and 36. Significance (FDR p-values) of GO term is color labeled from red to white. Similar GO terms are linked by edges, where the degree of similarity represented by the line width. (A) Module 13, (B) Module 36.

All up-regulated genes identified from pairwise comparisons belonged to either module 13 or 36 (Table 7). By performing WGCNA, additional relationships of these genes with other important biological mechanisms were identified. Module 13 consisted of multiple independent primary metabolisms with a major cluster of nitrogen compound metabolism connected with biological processes such as transcription regulation and homeostatic process. Module 36 related to RNA metabolism and biosynthesis with protein metabolism, phosphorylation, post-translational protein modification and homeostatic process (Fig 3). These complex inter-relationships among biological processes will be useful crowding stress tolerance networks influencing sweet corn crop yield.

### Transcription factor

Module 10 and 32 were clusters of genes not identified from pairwise comparisons, because expression of genes showed up-regulation only in H2 and H3, and in H1 and H3, respectively (S3 Fig). Yet, strong up-regulation of these modules in two of three high-yielding hybrids may indicate an important role in crowding stress tolerance. Module 10 has an over-represented function of cellular component organization. The plant genome is packaged by coiling to histone, creating the nucleosome, and further condensed to chromatin. Genome packaging is a crucial system for gene expression and very responsive to stress factors such as heat shock and oxidative stress by acting as a transcript regulator [77–81]. Chemical modifications, such as histone acetylation or methylation, directly affect chromatin structure changes by loosening or condensing the structure to alter gene transcription. Since chromatin modification is influenced by abiotic stress and modification impacts gene expression, the role of chromatin modification in stress response has been discussed extensively [82, 83]. For example, acetylation of histone H4 increased in response to cold, high salinity, and ABA application in *Arabidopsis* and tobacco cells [84]. Expression of histone protein, H2B and H4, was involved in module 10 indicating up-regulation of these genes might be involved in crowding stress tolerance in sweet corn.

The most over-represented biological function in module 32 was transcription regulation. Two bZIP transcription factors, bZIP71 and bZIP118, belonged to module 32. Expression of bZIP71 in rice was associated with drought, polyethylene glycol, salinity, and ABA applications [85]. Auxin responsive protein IAA17 also belonged to module 32. Salt stress condition reduced root elongation by increasing IAA17 stabilization and repressing auxin signaling in *Arabidopsis* [86]. These transcriptional factors may interact closely to confer crowding stress tolerance in sweet corn. One of *Arabidopsis* bZIP family, bZIP11, was associated with histone acetylation to influence the expression of auxin responsive genes such as IAA proteins [87]. Therefore, the interaction among these transcriptional factors in module 10 and 32 would be an important crowding stress tolerance mechanism in sweet corn. Further investigation on exploiting the interaction may be helpful.

## Conclusion

Sweet corn productivity can be improved with greater utilization of crowding stress tolerance. The most useful mechanisms of crowding stress tolerance will be those influencing crop yield. Genomic information from this research identified several potential crowding stress tolerance mechanisms linked to yield improvement. Gene expression patterns revealed each high-yielding hybrid had unique mechanisms to tolerate crowding stress. However, we also identified networks of related genes associated with crowding stress tolerance. Specifically, genes involved in biological functions such as photosynthesis, glycolysis, cell wall, carbohydrate/nitrogen metabolic process, chromatin and transcription regulation related processes were identified as possible mechanisms of crowding stress tolerance. Crowding stress tolerance genes were inter-connected with many biological functions, indicating complex nature of crowding stress tolerance mechanisms and the importance of improving mechanisms as a network.

## Supporting Information

S1 Fig Heatmap of 7,670 genes that had a one-way ANOVA FDR p-value<0.05 and at least 1.5 FC between any two hybrid comparisons. (PDF)

S2 Fig PCA plot of hybrids in respect to PC1 and PC2 using normalized probe expression values. (PDF)

S3 Fig Bar plots of mean eigengene values (+/- 1 SEM) of each hybrid for the modules with significant expression difference between yield groups. (PDF)

S1 File Genes and primers used for RT-qPCR validation. (PDF)

S2 File Microarray result and RT-qPCR validation of selected transcripts. (PDF)

S3 File Leaf nitrogen and LAI of sweet corn hybrids and yield groups measured in a field experiment near Urbana, IL in 2013. (PDF)
