# Supplementary material for: Identification of Crowding Stress Tolerance Co-Expression Networks Involved in Sweet Corn Yield
Source: PLoS One. 2016 Jan 21;11(1):e0147418. doi: 10.1371/journal.pone.0147418 (PMC4721684; doi:10.1371/journal.pone.0147418)

S1 Fig. Heatmap of 7,670 genes that had a one-way ANOVA FDR p-value<0.05 and at least 1.5 FC between any two hybrid comparisons

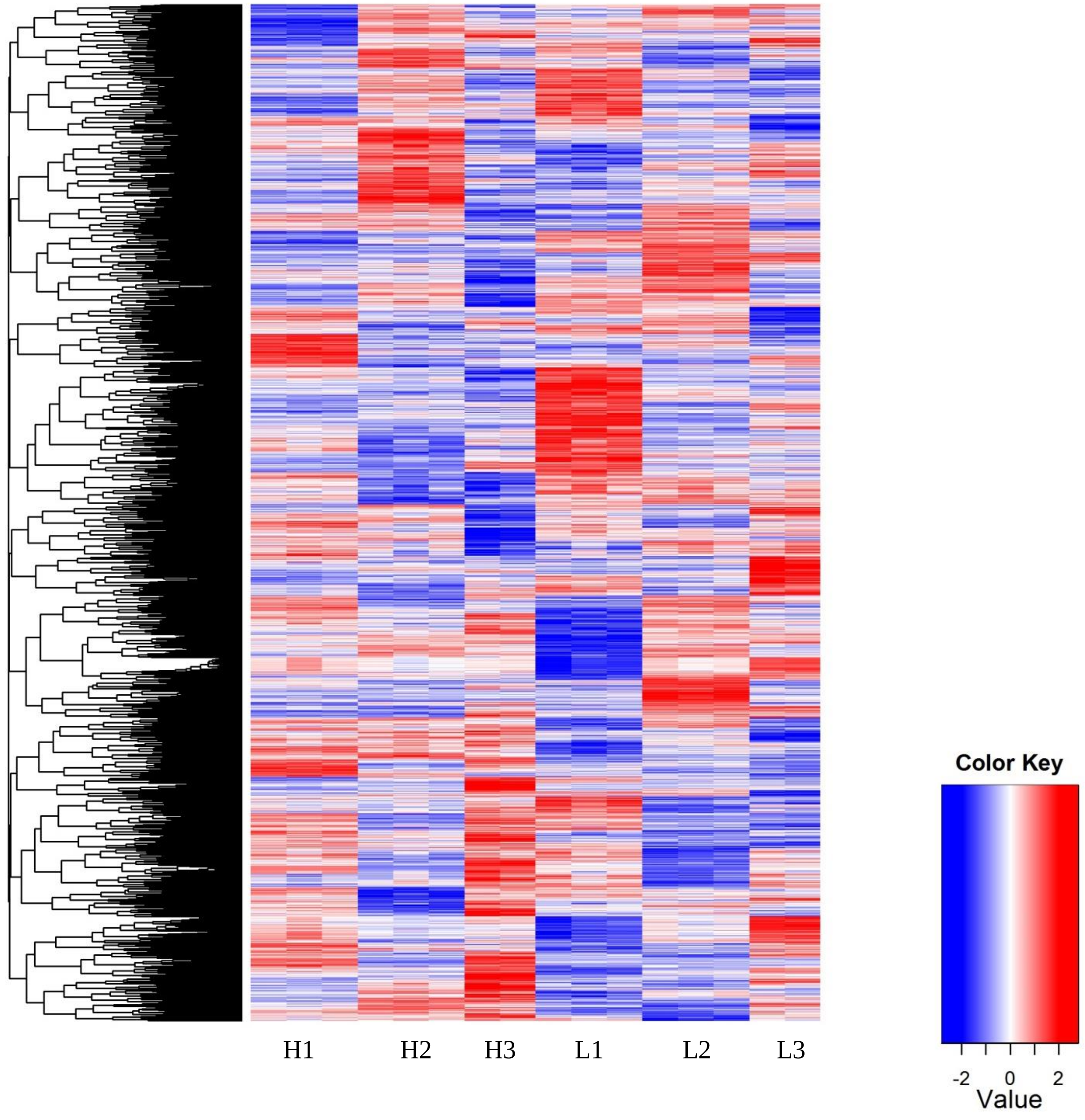

Supplement: S1 Fig — (PDF) [file pone.0147418.s001.pdf]
