# Supplementary figures and images for: Identification of Crowding Stress Tolerance Co-Expression Networks Involved in Sweet Corn Yield
Source: PLoS One. 2016 Jan 21;11(1):e0147418. doi: 10.1371/journal.pone.0147418 (PMC4721684; doi:10.1371/journal.pone.0147418)

S2 Fig. PCA plot of hybrids in respect to PC1 and PC2 using normalized probe expression values.

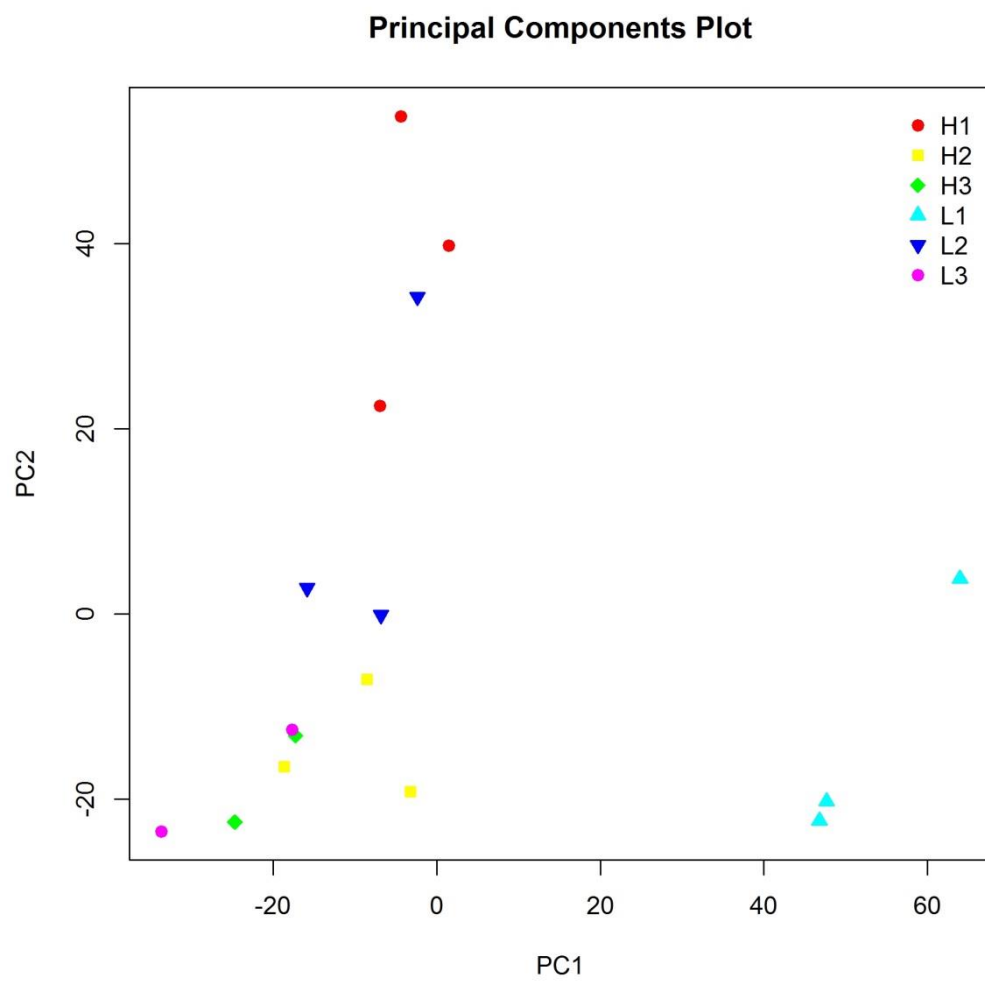

Supplement: S2 Fig — (PDF) [file pone.0147418.s002.pdf]
