# Supplementary material for: Identification of Crowding Stress Tolerance Co-Expression Networks Involved in Sweet Corn Yield
Source: PLoS One. 2016 Jan 21;11(1):e0147418. doi: 10.1371/journal.pone.0147418 (PMC4721684; doi:10.1371/journal.pone.0147418)

S3 Fig. Bar plots of mean eigengene values (+/- 1 SEM) of each hybrid for the modules with significant expression difference between yield groups. Positive and negative eigengene values represent up-regulation and down-regulation gene expression patterns in corresponding hybrid. (A) Module 22 showed the most significant difference between yield groups (FDR P-value= 0.001). The genes in module 22 had down-regulation in all high-yielding hybrids and up-regulation in all low-yielding hybrids. (B) Module 36 showed up-regulation in all high-yielding hybrids and down-regulation in all low-yielding hybrids (FDR P-value= 0.003). (C) Module 13 showed up-regulation in all high-yielding hybrids and down-regulation in low-yielding hybrids except L3 (FDR P-value= 0.006). (D) Module 32 showed up-regulation in H2 and H3 and down-regulation in all low-yielding hybrids (FDR P-value= 0.017). (E) Module 38 showed up-regulation in all high-yielding hybrids and down-regulation in L2 and L3 (FDR P-value= 0.017). (F) Module 9 showed down-regulation in all high-yielding hybrids and up-regulation in L1 and L2 (FDR P-value= 0.017). (G) Module 17 showed down-regulation in H2 and H3 and up-regulation in all low-yielding hybrids (FDR P-value=0.022). (H) Module 10 showed up-regulation in H1 and H3and down-regulation in all low-yielding hybrids (FDR P-value= 0.040). (I) Module 14 showed down-regulation in all high-yielding hybrids and up-regulation in L2 and L3 (FDR P-value= 0.043).

A. Module 22

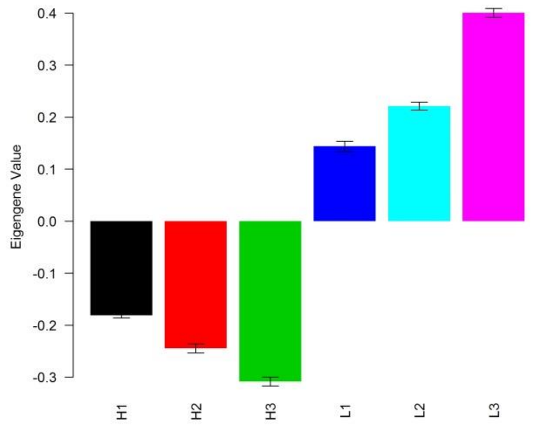

B. Module 36

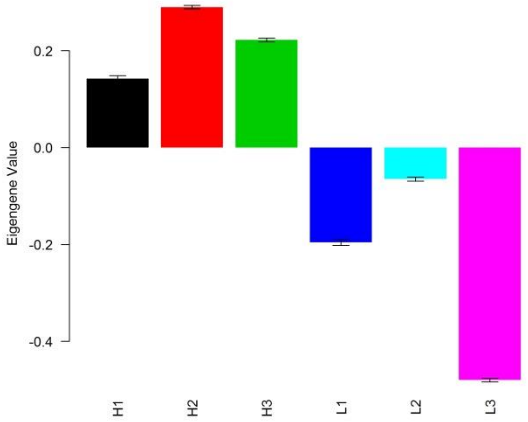

C. Module 13

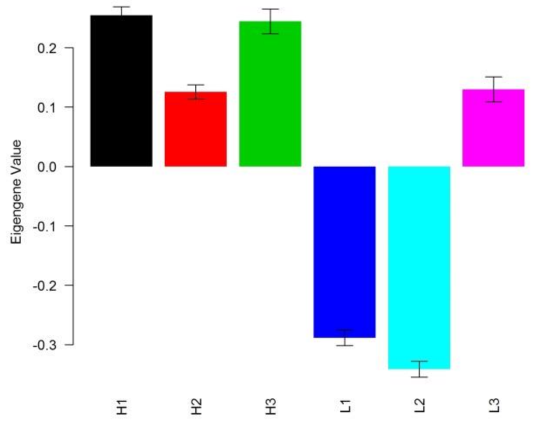

D. Module 32

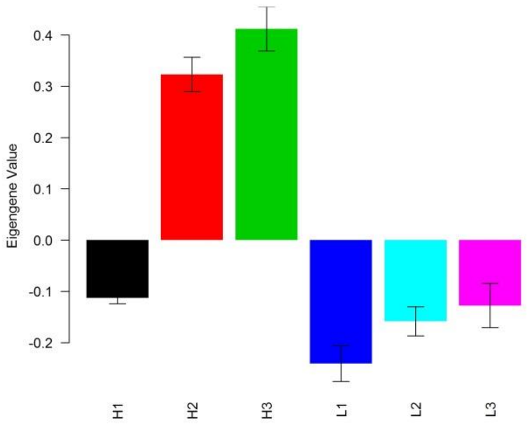

E. Module 38

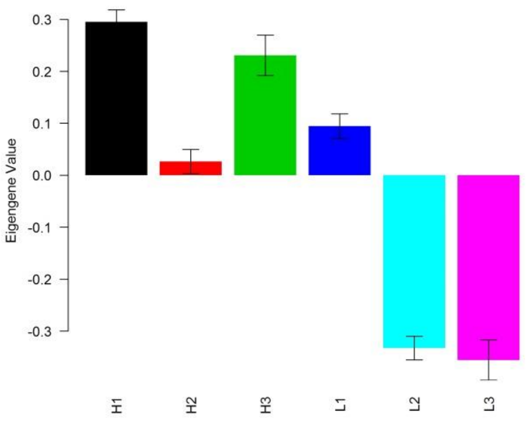

F. Module 9

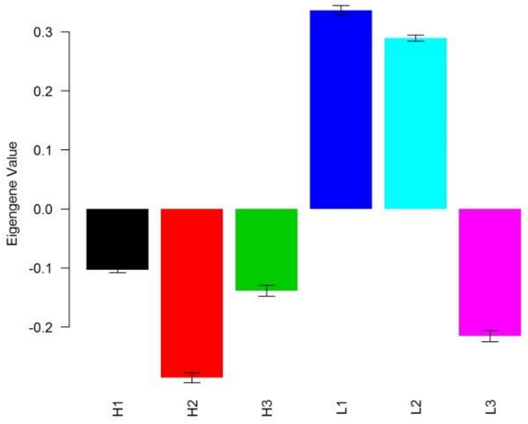

G. Module 17

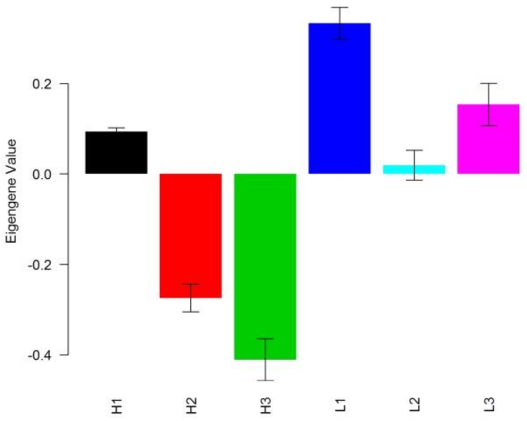

H. Module 10

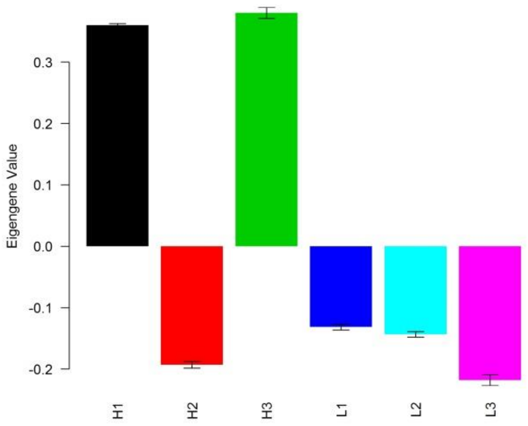

I. Module 14

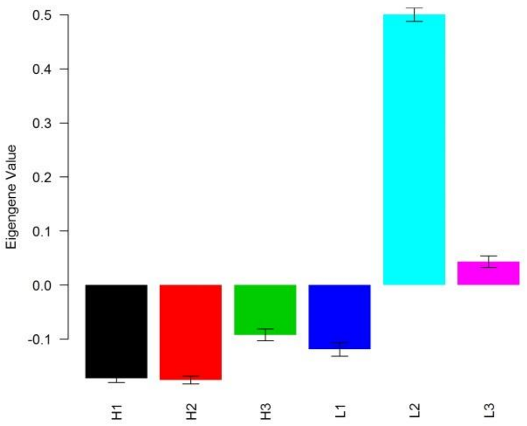

Supplement: S3 Fig — (PDF) [file pone.0147418.s003.pdf]
