# Supplementary material for: Identification of Crowding Stress Tolerance Co-Expression Networks Involved in Sweet Corn Yield
Source: PLoS One. 2016 Jan 21;11(1):e0147418. doi: 10.1371/journal.pone.0147418 (PMC4721684; doi:10.1371/journal.pone.0147418)
Supplement: S1 File — (PDF) [file pone.0147418.s004.pdf]

S1 File. Genes and primers used for RT-qPCR validation.

Transcript ID	Gene name	Type		Primer sequence
GRMZM2G018447 _T01	Ubiquitin conjugating enzyme	Control	Forward	AGTTGCAGGTTGACTTCCC
			Reverse	TGCTGGCGAACTAGACAAC
GRMZM2G124532 _T03	phyB1	Candidate	Forward	GGCATCCGATTTACCAACTCTC
			Reverse	CAACAGCATTCATAACGTCTCC
GRMZM5G854473 _T01	BolA-like protein	Candidate	Forward	CTCTCAGCCCTTCCAATCTC
			Reverse	GGATCTCCGCCATGTGAG
AC205100.3 _FGT001	glycolysis	Candidate	Forward	GCCTATTGCTCATTTTCTCAGG
			Reverse	AGTTTGATGTTTTGGTTGTGGG
GRMZM2G374302 _T02	Arginine decarboxylase	Candidate	Forward	CAGTTCCCCATGATCCTTCG
			Reverse	TCGAGCCCATAGTTCCATTG
