# Supplementary material for: Identification of Crowding Stress Tolerance Co-Expression Networks Involved in Sweet Corn Yield
Source: PLoS One. 2016 Jan 21;11(1):e0147418. doi: 10.1371/journal.pone.0147418 (PMC4721684; doi:10.1371/journal.pone.0147418)
Supplement: S2 File — (PDF) [file pone.0147418.s005.pdf]

S2 File. Microarray result and RT-qPCR validation of selected transcripts.

Comparisons	Expression	GRMZM2G124532_T03 (phyB1)	GRMZM5G854473_T01 (BolA-like protein)	AC205100.3_FGT001 (Glycolysis)	GRMZM2G374302_T02 (Arginine decarboxylase)
H1 vs L1	Microarray	-1.08[a] NS[b]	2.01 ****	7.88 ****	2.06 ***
	RT-qPCR	-1.28 NS	3.72 ***	24.95 ****	17.91 *
H1 vs L2	Microarray	-1.21 NS	2.15 ****	8.32 ****	1.90 **
	RT-qPCR	-1.73 NS	3.00 *	9.94 ***	7.69 *
H1 vs L3	Microarray	-1.16 NS	1.79 ***	8.11 ****	1.90 *
	RT-qPCR	1.05 NS	4.80 ***	24.74 ****	7.10 *
H2 vs L1	Microarray	1.07 NS	2.66 ****	9.93 ****	1.89 **
	RT-qPCR	-1.50 NS	2.88 ***	23.96 ****	16.92 NS
H2 vs L2	Microarray	-1.04 NS	2.84 ****	10.49 ****	1.74 *
	RT-qPCR	-2.03 NS	2.32 NS	9.54 **	7.27 NS
H2 vs L3	Microarray	1.00 NS	2.37 ****	10.22 ****	1.75 *
	RT-qPCR	-1.12 NS	3.71 ***	23.76 ***	6.70 NS
H3 vs L1	Microarray	-1.07 NS	2.19 ****	9.08 ****	3.04 ***
	RT-qPCR	-1.04 NS	2.07 ***	27.43 ****	38.53 ****
H3 vs L2	Microarray	-1.20 NS	2.34 ****	9.59 ****	2.80 ***
	RT-qPCR	-1.40 NS	1.67 NS	10.93 ****	16.56 ****
H3 vs L3	Microarray	-1.15 NS	1.95 ****	9.34 ****	2.81 ***
	RT-qPCR	1.29 NS	2.67 ****	27.20 ****	15.27 ****
H123 vs L123	Microarray	-1.09 NS	2.26 ****	9.18 ****	2.12 ***
	RT-qPCR	-1.26 NS	2.85 ****	18.64 ****	12.57 ****

[a] Fold change difference of gene expression. Positive and negative signs indicate the respective gene was up- or down-regulated in the first hybrid in the comparison, respectively.

[b] Fold change was significant when p-value <0.05, <0.01, <0.001 and <0.0001 labeled *, **, ***, and ****, respectively. Fold change not significant labeled NS.
