# Supplementary material for: Identification of Crowding Stress Tolerance Co-Expression Networks Involved in Sweet Corn Yield
Source: PLoS One. 2016 Jan 21;11(1):e0147418. doi: 10.1371/journal.pone.0147418 (PMC4721684; doi:10.1371/journal.pone.0147418)
Supplement: S3 File — (PDF) [file pone.0147418.s006.pdf]

S3 File. Leaf nitrogen and LAI of sweet corn hybrids and yield groups measured in a field experiment near Urbana, IL in 2013.

Factor	Effect	Leaf nitrogen	LAI
		%	-
**Hybrid**	H1	2.97 a[a]	5.00 a
	H2	2.77 a	5.13 a
	H3	2.88 a	5.83 a
	L1	2.93 a	6.48 a
	L2	2.85 a	5.77 a
	L3	2.95 a	4.96 a
**Group**	High	2.87 a	5.32 a
	Low	2.91 a	5.74 a

[a] Mean comparisons were performed among hybrids and between groups. For each effect, the same letters within a column indicate the means are not significantly different at α=0.05.
